# Enough is enough: salvage procedures in severe periprosthetic joint infection

**DOI:** 10.1186/s42836-023-00182-7

**Published:** 2023-07-03

**Authors:** Yves Gramlich, Javad Parvizi

**Affiliations:** 1 Department of Trauma and Orthopedic Surgery, Berufsgenossenschaftliche Unfallklinik Frankfurt Am Main, Frankfurt, 60389 Germany; 2 Rothman Orthopaedic Institute at Thomas Jefferson University, Philadelphia, PA 19107 USA

**Keywords:** Amputation, Arthrodesis, Antibiotic suppression, Debridement, antibiotics, implant retention, Periprosthetic joint infection, Salvage procedure

## Abstract

**Background:**

In severe cases of periprosthetic joint infection involving negative host-dependent factors, individual-based decisions between a curative therapy *vs*. salvage procedure are necessary. We aimed to review salvage procedures in severe periprosthetic joint infection cases, where a gold standard of a curative two-stage exchange can no longer be achieved. The options of knee arthrodesis, amputation, persistent fistula (stable drainage), or a debridement, antibiotics, and implant retention procedure in late-onset cases are discussed, including lifelong antibiotic suppression alone.

**Methods:**

We focused on known salvage procedures for severe periprosthetic joint infection of the hip and knee, such as amputation, arthrodesis, antibiotic suppression, persistent fistula, and debridement, antibiotics, and implant retention in late-stage infections, and the role of local antibiotics. The current literature regarding indications and outcomes was reviewed.

**Results:**

Whereas a successful single-stage above-knee amputation can be a curative effort in younger patients, this is associated with limited outcome in older patients, as the proportion who receive an exoprosthesis leading to independent mobility is low. Therefore, arthrodesis using an intramedullary modular nail is an option for limb salvage, pain reduction, and preservation of quality of life and everyday life mobility, when revision total knee arthroplasty is not an option. Carrying out a persistent fistula using a stable drainage system, as well as a lifelong antibiotic suppression therapy, can be an option, in cases where no other surgery is possible. Active clinical surveillance should then be carried out. A debridement, antibiotics, and implant retention procedure in combination with local degradable antibiotics can be used and is an encouraging new option, but should not been carried out twice.

**Conclusion:**

Whereas the gold standard in periprosthetic joint infection treatment of late infections remains the exchange of the prosthesis, salvage procedures should be considered in the cases of reduced life expectancy, several recurrences of the infection, patients having preference and negative host factors. In these cases, the appropriate salvage procedure can temporarily lead to remission of the infection and the possibility to maintain mobility.

## Background

The number of endoprosthetic procedures is increasing annually, with a concomitant rise in the number of revision surgeries. The projected volume of primary and revision total knee arthroplasty will pose an immense burden on future health care systems over the next 30 years [1]. Compared to primary arthroplasty, the treatment cost of two-stage implant replacements increases, on average, by a factor of 3.4–6 [2]. Nevertheless, especially difficult-to-treat and chronic cases call for two-stage or multi-stage revision arthroplasty, which reportedly had high remission rates [3].

Apart from the financial challenge, some complicating factors, including modular implants and multidrug-resistant pathogens, require interdisciplinary efforts, thus resulting in organizational and professional challenges. Radical debridement plays an essential role in surgical treatment aimed to prevent postoperative reinfections, though bone stock and muscular tissue preservation to facilitate re-implantation and improve functional outcomes have been given more attention to the latter [4]. After the removal of the prosthesis, poly-methyl-methacrylate (PMMA) spacers are most commonly used for dead space management and local delivery of antibiotics [5–7]. Depending upon the treatment algorithms, the condition of the patient, and the extent of the infection, the time from resection arthroplasty to re-implantation varies from two weeks to several months [8].

With the number of revision surgeries increasing, equally growing mortality rates represent a life-limiting issue for elderly patients and those with multiple morbidities [9]. The host grade, as defined by the Musculoskeletal Infection Society (MSIS), is a key determinant of remission in two-stage revision arthroplasty for periprosthetic knee infections. Indeed, MSIS type A hosts with acceptable wounds (MSIS type 1 or 2) achieved a 70% success rate whereas repeat two-stage arthroplasty failed in MSIS type C3 hosts [10]. Furthermore, mortality rates for two-stage revision arthroplasty in elderly patients over 80 years were reported to be up to 36.7% [9]. Thus, alternative salvage procedures for elderly patients and those with multiple morbidities using a minimal number of surgeries with comparable remission rates are needed.

In certain cases, according to specific criteria as published by the Infectious Diseases Society of America (IDSA), one-stage septic exchange arthroplasty is a viable treatment alternative [8, 11, 12]. However, only a limited number of cases meet the IDSA criteria, and for some patients, even single-stage exchange arthroplasty bears a high risk due to age, multiple morbidities, and reduced bone stock. For these patients, adequate treatment options, including implant retention, have yet to be developed. Only in patients with suspected acute periprosthetic joint infection (PJI), may a debridement, antibiotics, and implant retention (DAIR) procedure offer high remission rates if its use strictly follows the diagnostic and therapeutic criteria [13, 14]. However, chronic or difficult-to-treat cases do not satisfy the published guidelines/criteria, and, consequently, DAIR procedures are not an effective therapeutic strategy [15].

Most problems encountered in complex revision total knee arthroplasty (TKA) can be managed with a wide range of implant systems currently available (e.g., modular metaphyseal sleeves, metal augments, or cones). High-constraint implants offer sufficient stability even in cases with extended ligamentous deficiencies. Even when the extensor mechanism fails, an arthrodesis can prevent an amputation.

**Fig. 1:**
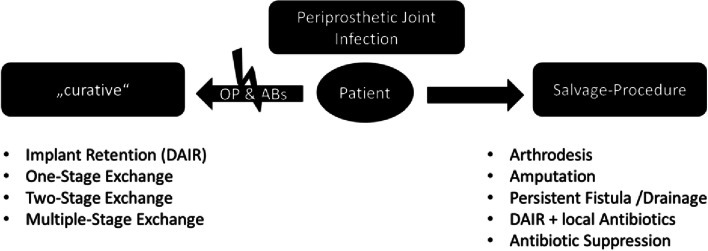
Flow diagram of decisions to perform a salvage procedure. The decision to perform a salvage procedure in the cases of PJI depends on the conditions of patients, who may be subjected to a curative operation (OP) or antibiotic therapy (ABs). If the patient does not fulfill the requirements for the gold-standard or curative treatment options, salvage-procedures should be considered

We aimed to review salvage procedures in severe cases of PJI, where a gold standard of a curative two-stage exchange can no longer be achieved. The options of knee arthrodesis, amputation, persistent fistula (stable drainage), or a DAIR procedure in late-onset cases are discussed, as well as a lifelong antibiotic suppression alone (Fig. 1). The role of modern local antibiotics is also discussed.

## Methods

We focused on known salvage procedures for severe periprosthetic joint infection of the hip and knee, such as amputation, arthrodesis, antibiotic suppression, persistent fistula, and debridement, antibiotics, and implant retention in late infections, as well as the role of local antibiotics. A review of the current literature indications, as well as outcomes, was conducted. Our literature research was limited to the aforementioned procedures. Original articles as well as current guidelines were included, such as international consensus meeting protocols [16] or guidelines of the European Bone and Joint Infection Society (EBJIS) [17].

### Indications for salvage-procedures

The current gold-standard of PJI treatment is the exchange of the implant using one-, two- or multiple exchange procedures [3], whereas the DAIR procedure is an evidence-based strategy involving debridement, antibiotic therapy, and implant retention for acute PJI [18–20]. It is known that the successful treatment is based on the combination of sufficient radical debridement and (biofilm targeting) antibiotic administration for a specific duration [3]. If the patient is not suitable for either the curative radical surgical treatment or the antibiotic treatment, salvage-procedures should be considered (Fig. 1). In addition, patients informed consent and shared decision-making should be a must in complex cases of multimorbid or older patients, as the goal of the therapy may not be a curative eradication of the infection, since the goal for these patients is more often a short treatment period, rapid independent mobility and few side effects of the necessary antibiotic therapy. The host grade (MSIS) is known to be a key determinant of remission in two-stage revision arthroplasty for periprosthetic knee infections [10]. Furthermore, mortality rates for two-stage revision arthroplasty in elderly patients over 80 years were reported to be up to 36.7% [9]. Thus, alternative salvage procedures for elderly patients and those with multiple morbidities using a minimal number of surgeries and adjusted objectives are needed.

### Arthrodesis

There is no role for arthrodesis of the hip in PJI as girdlestone procedure can be performed. Arthrodesis of the knee should be considered to be of curative nature when extension mechanism is lost or as a salvage-procedure to avoid amputation or after multiple TKA replacements when a painful arthroplasty is expected. Owing to radical debridement, the outcomes of septic revision TKA are often impaired by compromised soft tissue and loss of bone stock. Permanent damage to the extensor mechanism, for example, to facilitate wound closure in cases where skin grafts are often not possible, can even render TKA impossible. Difficult conditions foster the development and use of highly constrained implants to address ligamentous deficiencies and bone defects [21]. Metal augments, modular metaphyseal sleeves, or cones compensate for bone defects and achieve sufficient zonal fixation.

When severe ligamentous deficiencies are present, a (rotating) hinge prosthesis can be successfully used. Frequent complications are re-infection and prolonged wound-healing. Functional outcomes, pain levels, and pain-free walking distance are significantly lower compared to primary TKA [22, 23]. Accordingly, remission rates of only 61.6% after failed two-stage exchange, and re-implantation rates of only 65% following repeated debridement, have been reported [24]. Thus, successful joint reconstruction with currently available revision TKA systems is often precluded [25]. In these situations, amputation can be avoided by utilizing arthrodesis.

**Fig. 2:**
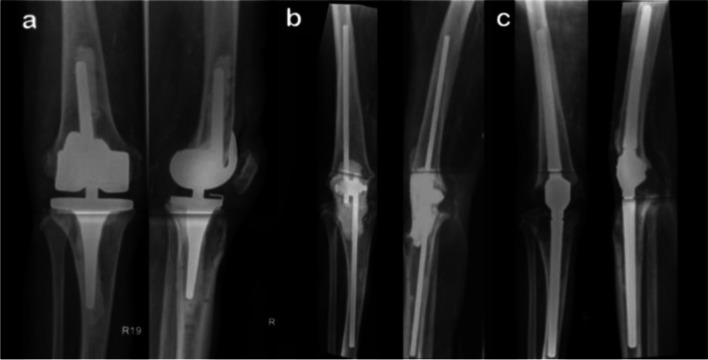
**a** X-rays of a patient with a hinged cemented total knee arthroplasty. **b** X-rays after prosthesis explantation, various revision stages and debridements, which led to extensor insufficiency and bone loss, as well as patellectomized with fixed spacer. **c** The joint reconstruction is carried out by implanting a distance arthrodesis prosthesis (Knee Arthrodesis Module, Brehm, Germany)

Limb preservation using arthrodesis can be achieved with several procedures, relying on different prerequisites. Therefore, the individual conditions of each patient have to be considered in treatment planning. The most common indications for knee arthrodesis are septic complications following TKA [25–28]. Fröschen et al. proposed the use of custom-made arthrodesis modules to achieve knee arthrodesis in cases of extensor mechanism failure, and concluded that arthrodesis improves leg function and reduces pain compared to surgeries using only revision prostheses [29]. Complete prosthesis removal often leads to extensive bone loss. In these situations, a contact arthrodesis, such as external fixation or screw-based arthrodesis, are not a feasible option. Hence, a distance arthrodesis is needed in the case of long-distance bone stock loss [25, 29]. These implants utilize intramedullary stems coupled with an arthrodesis module (Fig. 2).

Fixation of the arthrodesis module can be achieved using cemented or uncemented stems. The type of fixation remains controversial, especially in the cases of stem re-implantation following septic revision TKA or arthrodesis exchange. In TKA, cemented fixation of the femoral and tibial joint components is common, with an increasing number of studies reporting comparable results using cementless intramedullary stem fixation. However, studies reporting larger case series including septic revisions are lacking. In knee arthrodesis, advantages of cemented intramedullary stems are similar to those of revision TKA: lower periprosthetic fracture rate in the cases of reduced bone quality (osteopenia, osteoporosis); and reduced postoperative pain (including stem pain). Nevertheless, severe perioperative complications were reported in up to 45% of revision surgeries [30]. The higher loosening and revision rates of cemented implants can be attributed to the specific characteristics of TKA revision. Compared to primary TKA, a > 80% cement bone interface shear strength reduction is observed in revision arthroplasty [31]. Despite previous research efforts, an established standard regarding multistage arthroplasty revisions, including the use of spacers and the anchorage principles of the revision implant, have yet to be found [30].

A large cohort study matched 52 patients with arthrodesis with 52 patients with a modular revision implant after a multistage exchange following PJI of the knee [32]. Remission rates were comparable between the groups, reporting comparable pain reduction and improvement in overall quality of life. However, walking distance and activity and functional scores were significantly lower in the arthrodesis group than in the revision TKA group. Hence, arthrodesis using an intramedullary nail is a viable option for limb salvage, offering pain reduction and improvements in quality of life in patients when revision TKA is not an option [32].

Conversely, Röhner et al. reported that arthrodesis had an unsatisfactory outcome when performed after septic failure of revision TKA [33]. Nevertheless, the infection control rate was comparably low, with an up to 50% rate of reinfections and high-level postoperative pain. Arthrodesis, as well as a revision TKA, requires a high infection control rate before implantation to achieve considerable results.

### Amputation

With improvements in therapeutic strategies, and introduction of new-generation modular implants, and more effective antibiotic agents and delivery systems, the overall frequency of lower extremity amputations decreases. Alarmingly, a study conducted between 1998 and 2016 documented an increase in the frequency of hip disarticulation for PJI [34]. Given the financial burden of septic revision arthroplasty, patients without private insurance are at significantly higher risk of hip disarticulation. Hip disarticulation remains a procedure with high mortality and unfavorable outcomes, and even restricted ability to sit. A Girdlestone or other salvage procedure is recommended [35]. Contrastingly, above-knee amputation can be an option because patients with a failed two-stage exchange arthroplasty who undergo a repeat two-stage exchange arthroplasty demonstrate poor outcomes. Age, comorbidities (MSIS host grades), as well as bone stock and soft tissue status (MSIS extremity grades) should be considered when deciding whether an arthrodesis or an amputation should be performed. Despite the above-mentioned findings showing a good quality of life after the arthrodesis, other researchers reported good results achieved with amputation in combination with an exoprosthesis.

In these cases, amputations have to be carried out so that remission of infection can be achieved in a single-stage procedure, and revision of the amputation should be avoided. This remains a technical challenge because, on the one hand, for sitting and walking ability (using an exoprosthesis), the amputation should be performed a minimal distance from the knee; on the other hand, a safe, infection-free result can only be achieved with a safe distance between the amputation level and the PJI origin.

Hungerer et al. concluded that amputation, after failed multiple septic prosthesis changes in physically and mentally strong patients, is advantageous if they can be treated postoperatively with microprocessor-equipped exoprostheses [36]. A modern exoprosthesis can, therefore, lead to a high quality of life in well-selected patients, and surpasses an arthrodesis or prosthetic revision. The good functional result of amputees can be attributed to a lower average age of 63 years compared to the prosthesis group (69 years), and the age at amputation, according to Hungerer et al., influences the subsequent functional outcome considerably (80 years). However, these results cannot be extrapolated to patients in other studies, whose average age was well above 70 years. Well-selected younger patients could benefit from amputation and the fitment of a microprocessor-guided exoprosthesis over arthrodesis. However, the advantages of arthrodesis come into play with increasing age and previous illnesses, being independent of a highly selected group of patients. Good clinical and functional results can be achieved. Therefore, the result of an older individual suffering a recurrent PJI seems to not be the same as younger traumatic patients undergoing an amputation. Several studies pointed out the restricted outcome of amputations in PJI cases. In a recent study, patients who underwent above-knee amputation (AKA) for PJI had a low-level independence and ability to ambulate, in line with a high mortality rate [37]. Other studies reported similar low functional status in living patients with an AKA after infection, with only half of the patients walking after AKA [38]. Son et al. investigated factors that influence the decision between arthrodesis and AKA and found that high-volume arthroplasty centers with experienced clinicians more aggressively attempted to preserve the knee, even in the face of chronic PJI [39]. Although salvage procedures might be reduced, multistage revisions are, in turn, associated with a greater risk of subsequent knee arthrodesis or AKA. Despite the fact that AKA has a lower functional outcome and a higher mortality rate than arthrodesis [35], surgeons should be aware that outcomes of amputation versus arthrodesis depend on host grade and, therefore, the decision remains shared between the patient and surgeon.

### Persistent fistula or drainage

**Fig. 3:**
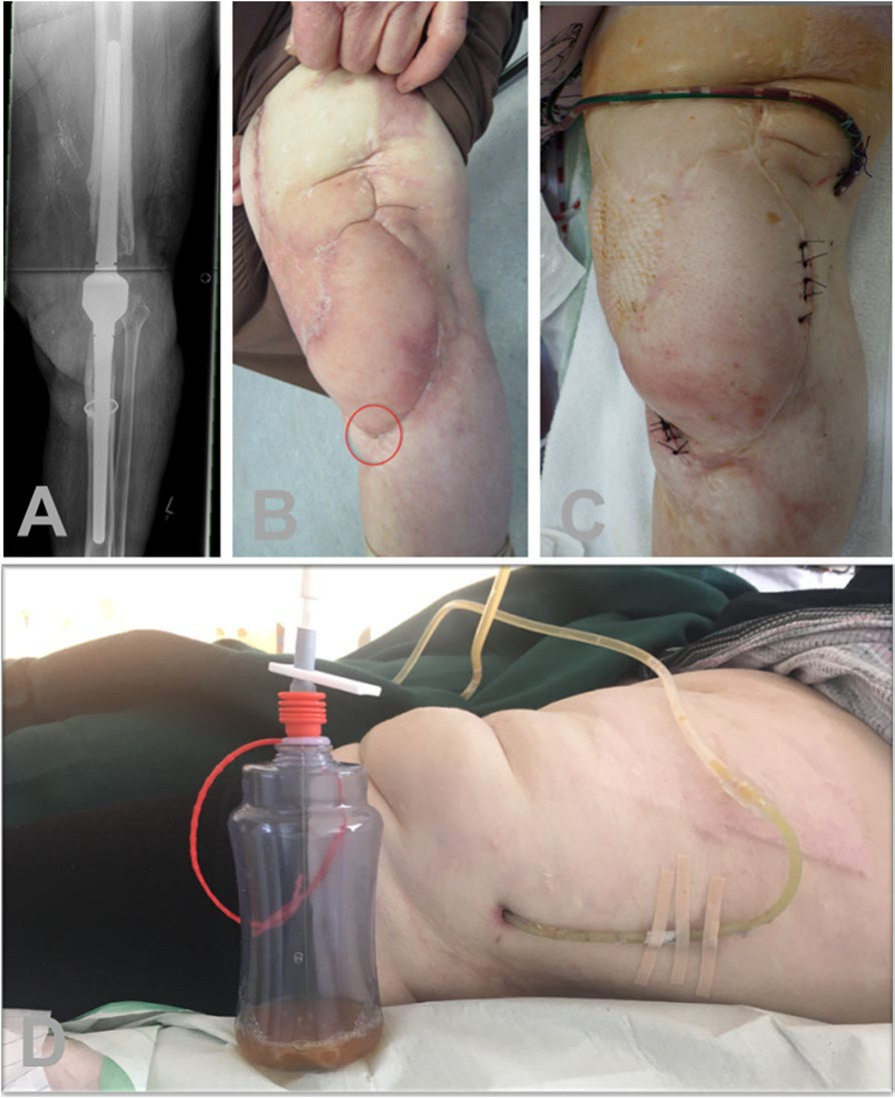
Images of a patient who underwent a curative arthrodesis of the left knee. **a** This was after multiple septic debridements and plastic surgery using a free flap. **b** After six years, a chronic infection in line with cutaneous fistula is present. **c** Accordingly, a persistent fistula using a 16 CH drainage tube was performed. **d** Patients regularly have to undergo maintenance of the drainage system

A persistent fistula (PF) is meant to be the last option in limb preservation, when even a curative AKA or a curative resection arthroplasty of the hip is not possible due to the patient’s host grade and individual operability. The procedure is well established for hips and knees. A PF is described as a natural fistula achieved without an artificial drainage tube, leading to a persistent infectious drainage out of the cutaneous fistula with a bag applied to the extremity. In the experience of the authors, a cutaneous stable fistula is difficult to achieve and leads to a permanent irritation of the surrounding skin due to the persistent pus evacuation. Therefore, we use a 16 CH or 18 CH tunneled artificial surgical drain ending in a drainage bottle or bag which can be carried at the leg. The procedure can be carried out within 30 min under spinal anesthesia and is, therefore, a short and non-invasive one-stage salvage procedure (Fig. 3).

Due to the organizational and psychological burden of PF, the indications thereof should be comprehensively discussed with each patient. Troendlin et al. reported a rejection rate of > 80% after a prolonged and vexatious course of illness [40]. PF is often performed to achieve faster convalescence and reduce further hospitalization, and such expectations often cannot be satisfied as, on average, three additional revision surgeries are needed after the PF was established [40]. The most common complication in patients with PF is closure of the fistula, with a potentially fatal course of the disease leading to sepsis [40]. The functional outcomes prior to and after PF are often identical and revision arthroplasty with implant removal has poorer functional outcomes after surgery compared to preoperation. A recent study of 159 patients with periprosthetic infection of the hip and knee, reported a poor functional outcome, with a Harris hip score (HHS) of 38.1 after PF, compared to 17.6 after Girdlestone resection [40]. Charlton et al. reported that the functional outcome after PF in periprosthetic infection of the hip was comparable to that after Girdlestone resection arthroplasty, with an HHS of 40 [41]. Patients with a PF at the knee scored a Knee Society Score of 34.1 [40] and, in fact, a recent study reported salvage procedures of the knee yielded much poorer outcomes than knee arthroplasties or arthrodesis [32].

In conclusion, a PF can be an option if the patient refuses any other salvage procedure, as the operation is short and feasible, even with bad host-dependent factors. However, the functional outcome is poor, and the drainage system requires regular maintenance, otherwise, the revision rates are high.

### Antibiotic suppression

Antibiotic suppression as a salvage therapy is not well defined, as different studies describe limited antimicrobial prophylaxis after DAIR or two-stage exchange, whereas others show lifelong administration of an antibiotic without any prior surgical therapy. We focus on the lifelong administration of an antibiotic in the cases of salvage procedures to prevent sepsis.

The role of suppressive antibiotics instead of surgical intervention for patients with chronic PJIs has not been investigated. Antibiotic suppression has the highest success rate after attempted surgical eradication, as the bacteria concentration is directly reduced. Chronic antibiotic suppression could then be regarded as a reasonable treatment option for select patients with persistent infection or multiple comorbidities. In this case, well-tolerated, orally available antibiotics (e.g., cotrimoxazole, doxycycline, or clindamycin) are administered in a resistogram-adapted manner over months to years. However, a recurrence of the infection must be expected after discontinuation of the therapy. The dose of the antibiotic can be partially reduced to 50% of the maximal daily dose in suppression therapy. A recent study [42] described antibiotic suppression as a reasonable strategy in PJI patients who lacked or refused further surgical treatment options: most hips and gram-positive infections were treated successfully and reoperations were avoided [42]. Focusing on streptococcal PJI, long-term oral antimicrobial suppression for at least six months was associated with significantly better outcomes as compared to no suppression (95% vs. 53%) in a non-homogenous cohort of patients receiving different initial surgical approaches [43]. Pavoni et al. [44] used antimicrobial suppression for PJI without surgery in 34 patients and they found no relapse in 17 patients. However, all patients received initial parenteral antimicrobial treatment, and the follow-up time was not consistent, ranging from 9 to 57 months. A recent study reported a series of 21 patients, without prior surgical intervention [45] and the two-year event-free survival rate was 40%. Overall, 11 patients were alive at the two-year follow-up. The follow-up period varied considerably, ranging from 1.3 to 56.5 months [45].

Whether long-term antibiotic suppression therapy should be considered instead of surgical intervention in patients with chronic PJI remains controversial. Delegates of an international consensus meeting in 2018 [16] stated that long-term suppressive oral antibiotics instead of surgical treatment may be considered for patients who are not eligible for surgery, when surgery is not expected to improve the functional outcome for a patient, and for patients who refuse surgery.

### DAIR as a salvage procedure and the role of local antibiotics

The DAIR procedure is an evidence-based strategy, involving debridement, antibiotic therapy, and implant retention in early PJI [18–20]. For acute PJI, high eradication rates were reported [13, 14]. Once a mature biofilm is present on the prosthesis, irrigation, exchange of mobile parts, and antibiotic therapy are not sufficient to eradicate the infection. Therefore, the DAIR procedure is not effective in chronic or difficult-to-treat cases [15], which do not comply with the criteria of recently released guidelines. Though a modified DAIR protocol seems promising, especially in the cases where exchange is not possible due to multiple morbidities or insufficient bone stock, results in published studies differed. A reason for the limited success in these cases is the modification in local circulation which prevents systemic antibiotics from reaching sufficient drug concentrations at the affected joint [46].

To address ineffective systemic antibiotic therapy, local application of antibiotics has evolved to an indispensable component of PJI therapy. PMMA has become the most widely used antibiotic carrier system, although an additional effect has not been proven [47, 48]. Moreover, the PMMA has to be surgically removed because the surface of the cement itself can act as an adhesive for bacteria and, therefore, foster biofilm formation and bacterial multidrug resistance, which limits its clinical utility [49, 50]. Furthermore, unfavorable release patterns and only partial elution limit the therapeutic value as an antibiotic carrier [51, 52]. Hence, interests in degradable antibiotic carriers which have favorable biological properties, sparing surgical removal and enabling complete antibiotic release, are on the rise [53–55]. To increase effect of the DAIR procedure on late-onset PJI, these degradable carriers should ensure sufficient antibiotic drug levels without the need for revision procedures in patients with multiple morbidities. In a recent study on late-onset cases, the DAIR procedures with and without topical calcium-based antibiotic carriers were evaluated. The combination of the DAIR procedure and local degradable antibiotics resulted in a significantly higher three-year infection-free survival [56, 57]. In addition to the biological properties, antibiogram-specific antibiotic loading increases the effectiveness. Despite these encouraging results, an exchange of the implants remains the gold standard, and that a DAIR in late infections is actually not recommended as a curative treatment [3, 8, 58]. According to the literature [59, 60], the success rate of the DAIR procedure in elderly, multimorbid patients with late-onset PJI has been currently improved from 18.2% to 65.2% with the use of antibiogram-based degradable antibiotic carriers [56]. In 2022, a study that focused on PJI of the hip showed promising results when additive degradable antibiotics were used [61]. Due to a significant paucity of data related to the usage of the DAIR procedure regarding applications outside the published criteria (e.g., as a salvage procedure in chronic PJI cases), recommendations vary and reported success rates ranged from 28 to 62% [59]. In a study by de Vries et al., higher infection-free survival was reported for patients with acute PJI compared to late-onset PJI (84% vs. 46.6%) [62]. Lora-Tamayo et al. [15] conducted the largest case study involving 463 cases of the DAIR procedure and the largest reported series of streptococcal PJI cases managed by DAIR to date. Results showed a worse prognosis than expected, with a failure rate being at 42.1% [55]. As a result, the use of the DAIR procedure for late-onset PJI cases is not recommended by the 2018 ICM criteria [60]. Nonetheless, the DAIR procedure, in combination with local antibiotics, caused no deaths over the entire follow-up period of 36 months in another study [56]. Compared to the recommended two-stage revision with mortality rates up to 36.7% in elderly patients (80 years or above) [20], current literature does not show a straightforward therapeutic decision based on ICM criteria.

**Fig. 4:**
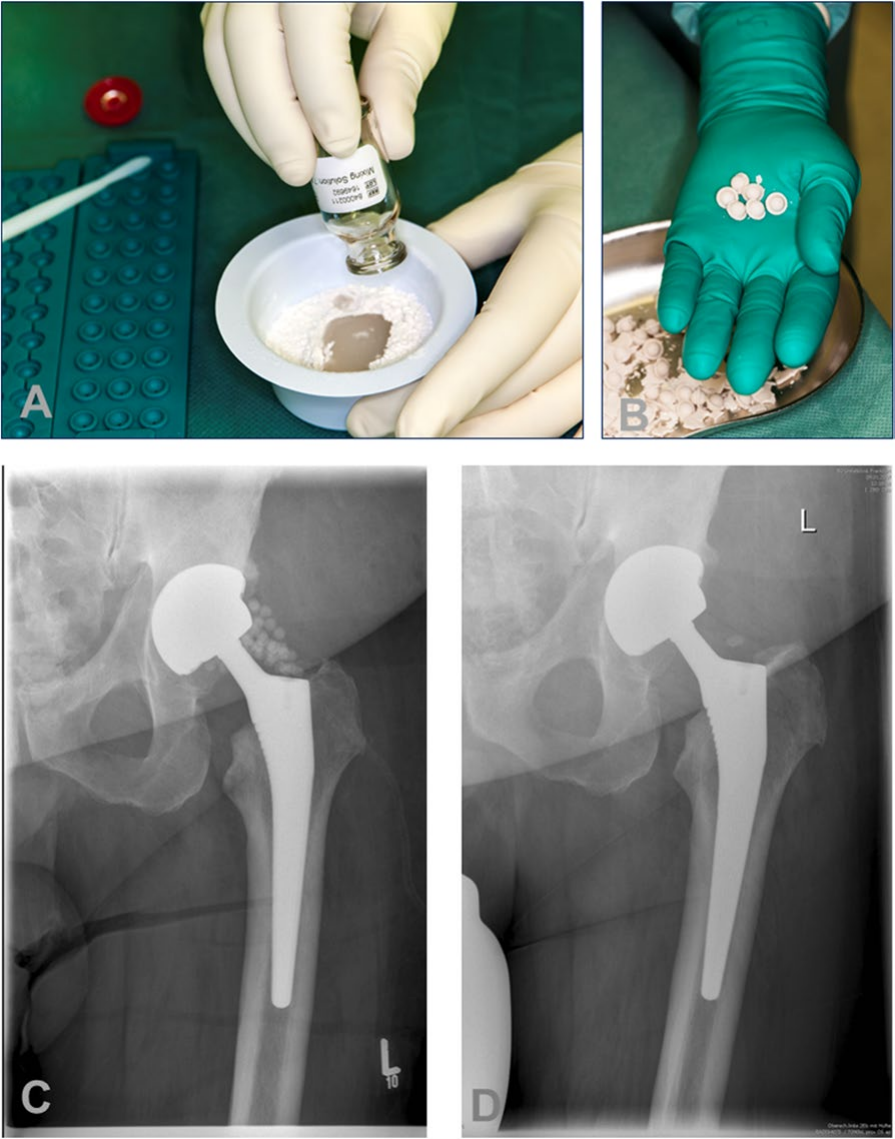
Bead application. **a** Application of the OSTEOSET (Wright Medical, USA) bead kit (admixed 3,000 mg of vancomycin per 30 beads). **b**, **c** Surgical single-stage application of beads in a periprosthetic joint infection of total hip arthroplasty during a debridement, systemic antibiotics, and implant retention (DAIR) procedure. **d** X-ray after one year shows degradation of the beads

Therefore, consciously used as a salvage procedure, the DAIR procedure, extended with degradable local antibiotics, can be a viable single-stage treatment option, when other procedures are not eligible (Fig. 4). In case of a failure of this treatment, a repeat DAIR is not recommended [60]. Further studies are required regarding this aspect of DAIR treatments.

### Specifics in antimicrobial therapy

All standard surgical procedures are based on a combination of radical surgical therapy to reduce the bacterial load and a highly effective antimicrobial therapy. Among a group of patients with microbiologically confirmed prosthetic joint infections that are managed with standard surgical procedures, antibiotic therapy for 6 weeks didn’t show non-inferiority to antibiotic therapy for 12 weeks and resulted in a higher percentage of patients with unfavorable outcomes [63]. The combination use of a biofilm-targeting antibiotic was shown to be able to improve outcomes, as rifampin is effective against the implant-associated infections caused by *Staphylococci* and *Propionibacterium spp*., whereas ciprofloxacin has anti-biofilm activity against gram-negative bacteria [64]. It should be taken into account that patients eligible for salvage procedures tend to have multiple comorbidities and other additional medications as well as higher ages. There is a special need for properly dosing and checking for interactions [65]. Specific particularities for geriatric patients must be taken into account. It is known, for example, that quinolones can cause dizziness, confusion and delirium in geriatric patients, but, on the other hand, have a very good anti-biofilm effect, whereas rifampin and linezolid are prone to enzyme induction. Antibiotic suppression has the highest success rate after attempted surgical eradication, since the bacteria concentration is directly reduced. Chronic antibiotic suppression could then be regarded as a reasonable treatment option for select patients with persistent infection or multiple comorbidities. In this case, well-tolerated, orally available antibiotics (e.g., cotrimoxazole, doxycycline, or clindamycin) are administered in a resistogram-adapted manner over months to years. However, a recurrence of the infection must be expected after discontinuation of the therapy. The dose of the antibiotic can be partially reduced to 50% of the maximal daily dose in suppression therapy. As the target of salvage-procedures is to provide mobility in the usual patient environment, antibiotic therapy should be provided as oral medication as soon as possible. Oral antibiotic therapy was non-inferior to intravenous antibiotic therapy when used during the first 6 weeks for complex orthopedic infection, as assessed by treatment failure at 1 year [66].

## Conclusions

The number of severely sick patients, who are too old for appropriate PJI treatment, is proposed to increase dramatically due to demographic changes. Overall, there is a lack of evidence regarding the indication, performance, and outcomes of salvage procedures. Recent studies showed the first evidence that arthrodesis could have advantages over amputation. Combining a DAIR procedure with modern calcium-based local antibiotics can lead to lower re-infection rates. Persistent fistulas using drainage systems or lifelong antibiotic suppression therapy should only be considered in specific cases, as the complication rates and outcome are not yet clear. Hip disarticulation remains a procedure with high mortality and unfavorable outcomes, and even the ability to sit is restricted. Performing a Girdlestone or other salvage procedure is recommended [35]. All patients should undergo active surveillance in experienced outpatient facilities that treat periprosthetic joint infections.

### Limitations

There is a lack of data and evidence in the field of salvage procedures for periprosthetic joint infection. Although the literature search in this article was made properly, most of the studies consisted of case series, retrospective research and consensus guidelines. Therefore, there can be no general treatment advice but clinicians may use these treatments in patients who are no longer suitable for standard procedures for PJI.

## Data Availability

Data sharing is not applicable to this article as no datasets were generated or analyzed during the current study.

## Abbreviations

AKA: Above-knee amputation
DAIR: Debridement, antibiotics, and implant retention
HHS: Harris hip score
IDSA: Infectious Diseases Society of America
MSIS: Musculoskeletal Infection Society
PF: Persistent fistula
PJI: Periprosthetic joint infection
PMMA: Poly-methyl-methacrylate
TKA: Total knee arthroplasty
